# Supplementary material for: Empirical methods for controlling false positives and estimating confidence in ChIP-Seq peaks
Source: BMC Bioinformatics. 2008 Dec 5;9:523. doi: 10.1186/1471-2105-9-523 (PMC2628906; doi:10.1186/1471-2105-9-523)
Supplement: Additional File 1 — A variety of html documents from the USeq web site detailing the available applications, their best usage, output file type descriptions, command line menus, etc. [file 1471-2105-9-523-S1.zip › USeqUserGuides/JavaDoc.zip!/JavaDoc/gata/aligner/class-use/GATAligner.html]

Uses of Class gata.aligner.GATAligner


|  |  |  |
| --- | --- | --- |
| **Overview** \| **Package** \| **Class** \| **Use** \| **Tree** \| **Deprecated** \| **Index** \| **Help** | |  |
| PREV   NEXT | **FRAMES**    **NO FRAMES**     **All Classes** |  |


---


## **Uses of Class gata.aligner.GATAligner**

| Packages that use GATAligner | |
| --- | --- |
| **gata.aligner** | For aligning and post processing DNA sequences using BLAST for subsequent import into GATAPlotter. |

Uses of GATAligner in gata.aligner

Constructors in gata.aligner with parameters of type GATAligner

`AlignerInputForm(GATAligner gata)`

`AlignerInputPanel(javax.swing.JFrame frame,
GATAligner gata)`
  
          Complex class containing most everything to show and process the GATAligner inputs.

---


|  |  |  |
| --- | --- | --- |
| **Overview** \| **Package** \| **Class** \| **Use** \| **Tree** \| **Deprecated** \| **Index** \| **Help** | |  |
| PREV   NEXT | **FRAMES**    **NO FRAMES**     **All Classes** |  |


---
